# Bioaccessible Antioxidants in Milk Fermented by *Bifidobacterium longum* subsp. *longum* Strains

**DOI:** 10.1155/2015/169381

**Published:** 2015-02-23

**Authors:** Mérilie Gagnon, Patricia Savard, Audrey Rivière, Gisèle LaPointe, Denis Roy

**Affiliations:** Institut sur la Nutrition et les Aliments Fonctionnels (INAF), Université Laval, 2440 Boulevard Hochelaga, Québec, QC, Canada G1V 0A6

## Abstract

*Bifidobacterium longum* subsp. *longum* is among the dominant species of the human gastrointestinal microbiota and could thus have potential as probiotics. New targets such as antioxidant properties have interest for beneficial effects on health. The objective of this study was to evaluate the bioaccessibility of antioxidants in milk fermented by selected *B. longum* subsp. *longum* strains during *in vitro* dynamic digestion. The antioxidant capacity of cell extracts from 38 strains, of which 32 belong to *B. longum* subsp. *longum*, was evaluated with the ORAC (oxygen radical absorbance capacity) method. On the basis of screening and gene sequence typing by multilocus locus sequence analysis (MLSA), five strains were chosen for fermenting reconstituted skim milk. Antioxidant capacity varied among the strains tested (*P* = 0.0009). Two strains of *B. longum* subsp. *longum* (CUETM 172 and 171) showed significantly higher ORAC values than the other bifidobacteria strains. However, there does not appear to be a relationship between gene sequence types and antioxidant capacity. The milk fermented by each of the five strains selected (CUETM 268, 172, 245, 247, or PRO 16-10) did not have higher initial ORAC values compared to the nonfermented milk samples. However, higher bioaccessibility of antioxidants in fermented milk (175–358%) was observed during digestion.

## 1. Introduction

Probiotic microorganisms, by definition, have proven their beneficial functionality for human health [1–3]. Within the large collection of microorganisms used in probiotic dairy products, bifidobacteria are interesting members, as they are natural inhabitants of the human gastrointestinal tract (GIT) and their presence has been associated with healthy colon microbiota [4, 5]. Although the diversity of colon microbiota changes dramatically throughout life [6],* Bifidobacterium longum* is an important inhabitant of both the infant and adult colon [7, 8], with* B. longum* subsp.* longum* representing the most common subspecies [7, 9].

Dairy products are widely used as a delivery mode for probiotics into the colon. However, to provide health benefits, the probiotics present in dairy products need to survive the harsh conditions of the GIT and arrive in the colon in sufficient quantities [10]. Bacteria passing the GIT are subjected to several stress conditions, such as stomach acidity and high concentrations of bile salts in the duodenum [11, 12]. As for most colon bacteria,* B. longum* is a strict anaerobe [13], so the presence of oxygen in the GIT (highest concentration at the beginning of the GIT) is an important additional stress factor with which this species has to cope. Oxygen, due to incomplete reduction, produces reactive oxygen species (ROS) that damage cellular macromolecules, for example, by breaking peptide bonds and inducing oxidation of membrane lipids [14]. Bacteria are known to have distinct mechanisms to protect themselves against oxygen. For instance, as for lactic acid bacteria [15–17],* B. longum* produces antioxidant molecules in order to scavenge free oxygen radicals [18]. However, not much information is available in the literature about this antioxidant capacity and its relation with the oxidative stress response in* B. longum*.

Several genes present in bifidobacteria encode proteins related to the oxidative stress response. Alkyl hydroperoxide reductase C (AhpC) is a NADH-oxidase homolog that reduces oxygen to hydrogen peroxide [13, 19, 20]. Complete genome sequencing of* B. longum* NCC2705 has revealed the presence of a gene (*trx*) encoding a thioredoxin reductase-like protein that is believed to cooperate with AhpC to eliminate hydrogen peroxide [4]. Other enzymes include ribonucleotide reductase alpha subunit (NrdA) and NTP pyrophosphohydrolase (MutT1) that are involved in DNA damage protection and repair after oxidative stress [19]. Moreover, polyphosphate granules (poly P) are formed in response to oxidative stress. The putative polyphosphate kinase gene (*ppk*) present in bifidobacteria is thought to be responsible for this poly P synthesis [21].

Oxidative stress also affects human health. Several diseases and disorders, such as inflammatory bowel disease [22, 23] and cardiovascular diseases [24], have been related to the presence of ROS. Improving the blood antioxidant status has been proposed as a way to reduce the occurrence of these diseases. Studies have demonstrated that a change in diet increases the antioxidant capacity of blood [25, 26]. For this, antioxidants present in the food matrix first need to be absorbed in the GIT and then utilized by human metabolism, which represents antioxidant bioavailability. Bioavailability is related to bioaccessibility which represents the ingested antioxidants that are available for absorption in the gut after digestion [27]. Several models have been used to study the bioaccessibility of antioxidants. One of these is the TNO* in vitro* model for digestion (TIM-1), which is a dynamic model for the upper GIT (stomach to ileum) [28–30]. Furthermore, this model can be used to evaluate survival of probiotics in the GIT [11, 31–33].

Within the* B. longum* species, several metabolic characteristics (such as the ability to degrade prebiotics [34]) display strain-dependent differences [35, 36], so antioxidant capacity should also be expected to differ among strains. The goals of this study were first to evaluate the antioxidant capacity of 32* B. longum* subsp.* longum* strains in order to link this capacity with the diversity of genes related to oxidative stress responses. Secondly, the bioaccessibility of antioxidants in milk fermented with five selected strains of* B. longum* subsp.* longum* showing a range of antioxidant capacities of milk was assessed using the TIM-1 model.

## 2. Material and Methods

### 2.1. Screening of* B. longum* subsp.* longum* Strains

#### 2.1.1. Bacterial Strains, Growth Conditions, and Viable Counts

The 32 strains of* B. longum* subsp.* longum* are listed in Table 1. For the ORAC assay, other bacterial strains than* B. longum* subsp.* longum* were used for comparison purposes, namely,* B. adolescentis* ATCC 15703,* B. breve* ATCC 15698,* B. catenulatum* CUETM 174,* B. longum* subsp.* suis* ATCC 27533,* B. longum* subsp.* infantis* ATCC 15702, and* B. animalis* subsp.* lactis* BB-12. The stock cultures were kept at −80°C in MRS broth supplemented with 20% (v/v) glycerol (EMD Chemicals, Fisher Scientific, Ottawa, ON, Canada). For each experiment, the strains were subcultured in MRS broth (Sigma-Aldrich, Oakville, ON, Canada) supplemented with 0.05% cysteine (Sigma-Aldrich) and 0.1% Tween 80 (Sigma-Aldrich) by adding 2% of the frozen stock. After 24 h of incubation at 37°C in a glove box anaerobic chamber (Plas-Labs Inc., Lansing, MI, USA), 1% of the first subculture was added to fresh medium and incubated for another 24 h at 37°C. After two subcultures, 1 mL of culture was centrifuged at 12,000 ×g for 10 min at 4°C. The pellet for DNA extraction was kept at −80°C. Also with the second subculture, 1% was added to 20 mL of MRS broth and incubated for 24 h. To determine viable counts, expressed as colony forming units (CFU), 0.1 mL of the appropriate dilution was added to molten MRS agar (MRS-based broth supplemented with 0.05% cysteine, 0.1% Tween 80, and 2% dextrose) by pour plating and incubated for 48 h at 37°C in a glove box anaerobic chamber containing an atmosphere of 80% N_2_, 10% H_2_, and 10% CO_2_ (Praxair, Quebec, QC, Canada). Dilutions for viable counts were performed with peptone water (1% of Bacto Peptone (BD Biosciences, Mississauga, ON, Canada) and 0.05% cysteine) with pH adjusted to 6.8.

#### 2.1.2. Oxygen Radical Absorbance Capacity Assay

The ORAC assay was performed on cell-free extracts in triplicate for each strain. Optical density at 600 nm of each culture was measured against MRS broth as blank with a VIS spectrophotometer Genesys 20 (Thermo Scientific, Waltham, MA, USA). Viable counts were carried out as described above. First, the 20 mL 24 h culture was centrifuged at 12,000 ×g for 10 min at 4°C. Then, the pellet was washed three times with 20 mL phosphate buffer (75 mM) and finally suspended in 20 mL of the same buffer. After incubating for 30 min at 37°C, cells were mechanically lysed with a XL-2020 sonicator (Misonix Inc. Farmingdale, NY, USA) at 50 watts, five times for 1 min with a cooling step on ice for 5 min between each sonication step. Next, to obtain the cell-free extract, lysed cells were centrifuged at 12,000 ×g for 10 min at 4°C. The supernatant (cell-free extract) was finally diluted in a 1 : 1 ratio with phosphate buffer. The ORAC assay was performed based on the technique described by Dávalos et al. [37], Saide and Gilliland [15], and Bazinet et al. [38]. The diluted cell-free extracts were analyzed in triplicate in a 96-well plate in the Fluostar Galaxy (BMG Labtechnologies, Durham, NC, USA). To each well, 200 *μ*L of fluorescein (Sigma-Aldrich) solution (0.036 mg/L), 20 *μ*L of diluted sample, and 75 *μ*L of 2,2′-azobis-2-aminopropane dihydrochloride (AAPH) (Sigma-Aldrich) solution (8.6 mg/L) were added. The ORAC assay quantifies the inhibition (expressed in percentage and time) of fluorescence produced by peroxyl radicals generated at a constant rate by thermal decomposition of AAPH. The antioxidant capacity is expressed in *μ*M Trolox Equivalent (TE) calculated from the Trolox (Sigma-Aldrich) standard curve.

#### 2.1.3. Multilocus Sequence Analysis

DNA extraction was performed with the DNeasy Blood & Tissue Kit: gram positive bacteria DNA extraction protocol (Qiagen, Mississauga, ON, Canada) with some modifications. To the lysis buffer 10 *μ*L/mL of 5 U/mL mutanolysin (Sigma-Aldrich) was added. Primers (see Supplementary Table S1 in the Supplementary Material available online at http://dx.doi.org/10.1155/2014/169381) were designed using Geneious Pro R6 software (Biomatters, San Francisco, CA, USA) based on the* B. longum* sequences available for each gene locus obtained from GenBank through the Geneious Pro R6 software. The PCR amplification volume of 50 *μ*L contained 1 *μ*L of DNA, 1 *μ*L of dNTP mix (10 mM), 2 *μ*L of each primer (10 mM), 5 *μ*L of 10X Taq buffer, 0.25 *μ*L of Taq DNA polymerase (Feldan, Quebec, QC, Canada), and 38.75 *μ*L of nuclease-free water. PCR amplification of the five genes for each strain was performed with a Tgradient (Biometra, Montreal Biotech, Montreal, QC, Canada) using the following program: one cycle at 94°C for 5 min, 30 cycles with denaturation at 94°C for 30 s, primer annealing at 58°C for 30 s, and DNA extension at 72°C for 30 s, and a final extension step at 72°C for 5 min. Next, DNA sequence analysis was carried out on both strands of the purified PCR products with the BigDye Terminator v3.1 cycle sequencing kit and 3100 Genetic Analyzer (Life Technologies, Burlington, ON, Canada).

The sequences of the forward and reverse strands were aligned using Geneious R6 software. The allele number for each distinct sequence variant was determined with nonredundant databases (NRDB) program (http://pubmlst.org/analysis/). Then a sequence type (ST) number was given to each distinct combination of alleles for the five genes with START2 software [39]. Finally, for each strain, individual gene sequences were concatenated and phylogenetic trees were built using Jukes-Cantor neighbor-joining with bootstrapping as statistical method.

### 2.2. Dynamic* In Vitro* Gastrointestinal Digestion of Fermented Milk

#### 2.2.1. Bacterial Strains and Growth Conditions

Five strains of* B. longum* subsp.* longum* (CUETM 172, CUETM 245, CUETM 247, CUETM 268, and PRO 16-10) were tested for their capacity to ferment reconstituted skim milk. The strains were subcultured in MRS-based broth (MRS without glucose; Rosell Institute, Montreal, QC, Canada) supplemented with 0.05% cysteine, 0.1% Tween 80, and 0.5% dextrose (EMD Chemicals) by adding 2% of the frozen stock culture. After 24 h of incubation at 37°C in a glove box anaerobic chamber, 1% of the first subculture was added to the MRS supplemented with 0.5% lactose (EMD Chemicals) instead of dextrose and incubated for 24 h at 37°C. After two subcultures as for the growth curves, 1% was added to 350 mL of reconstituted milk and incubated for 18 h at 37°C in a glove box anaerobic chamber. The milk was composed of 12% low heat skim milk powder (Agropur, Granby, QC, Canada), 0.6% yeast extract (BD Biosciences), and 2% dextrose. Yeast extract and dextrose were added to ensure optimal growth of the strains in milk.

#### 2.2.2. Dynamic* In Vitro* Digestion

The intake (300 g of fermented milk) was added to the TIM-1 (TNO Nutrition and Food Research Institute, Zeist, The Netherlands) and digested for 5 h at 37°C. TIM-1 run was performed as described by Fernandez et al., [31] which was based on Minekus et al. [40]. The fermented milk passed through four compartments connected in series to simulate the stomach, duodenum, jejunum, and ileum, separated by valve segments that were computer controlled. Description of gastric and ileal deliveries, initial contents, secretions, and dialysis fluid are provided in the Supplementary Material (Table S2). Before adding the fermented milk, initial contents and secretions were deaerated by bubbling nitrogen gas for 90 s. Throughout the digestion experiment, jejunal and ileal compartments and effluent were maintained under anaerobic conditions with nitrogen gas flow (Praxair). The container for ileal effluent was maintained on ice to prevent the multiplication of cells. Dialysis of the contents of jejunal and ileal compartments was performed with Purema polyethersulfone membrane (hollow fibres) Xenium 110 Dialyzer (Baxter, Deerfield, IL, USA).

#### 2.2.3. Survival Evaluation and ORAC Analysis

Bacterial growth was measured by viable counts as described above and by propidium monoazide treatment in combination with quantitative PCR with (PMA-qPCR). Samples were taken from fermented milk at the start and from the TIM-1 at the following points: 30 and 60 min from the gastric compartment, at 60, 120, 180, and 240 min from the duodenal compartment, at 300 min from the combined jejunal and ileal compartments, and at 60, 120, 180, 240, and 300 min from the ileal effluent. PMA treatment was carried out as follows. One mL of sample was mixed with 42.4 *μ*L of 50% (w/v) sterile trisodium citrate solution (BDH Chemicals, Toronto, ON, Canada) and centrifuged 12,000 ×g for 10 min at 4°C. Cell pellets were suspended in 500 *μ*L of 2X TE (20 mM Tris HCL pH 8.0, and 2 mM EDTA). PMA (Biotium, Hayward, CA, USA) was added to the samples at a final concentration of 50 *μ*M and the samples shaken in the dark for 5 min were placed in the PMA lamp apparatus (LED-Active Blue, Ingenia Biosystems, Barcelona, Spain) for 15 min. Finally, the PMA-treated cell suspensions were centrifuged 12,000 ×g for 10 min at 4°C and the cell pellets were stored at −80°C until DNA extraction.

DNA extraction was performed based on the protocol of Licitra et al. [41]. Briefly, the DNeasy Blood & Tissue Kit: gram positive bacteria DNA extraction protocol was used with some modifications. The cell pellets were suspended in 400 *μ*L (for milk and stomach samples) or 180 *μ*L (for other samples) of enzymatic lysis buffer (20 mM Tris HCl pH 8.0, 2 mM EDTA, 1.2% Triton X-100, 20 mg/mL lysozyme (Sigma-Aldrich), and 10 *μ*L/mL of 5 U/mL mutanolysin (Sigma-Aldrich)) and incubated at 37°C for 1 h. Next, 25 *μ*L of proteinase K and 200 *μ*L of AL buffer were added and incubated at 70°C for 30 min. The suspensions were transferred to 2 mL microtubes containing 0.3 g of 1 mm diameter zirconium beads (Biospec Products, Bartlesville, OK, USA) and shaken twice for 90 s in a Mini-BeadBeater-16 (Biospec Products). Then, samples were centrifuged at 10,000 ×g for 10 min. Finally, 200 *μ*L of ice-cold absolute ethanol was added and DNA purification was performed according to the Qiagen protocol. The samples were stored at −20°C until qPCR amplification.

DNA quantification was performed with Applied Biosystems 7500 Fast Real-Time PCR System with software version 2.0.1 (Life Technologies). Primers tuf_F (5′-ACCTGGCCACGCTCGACATC-3′) and tuf_R (5′-AGACCATGGACGCCTGCGAG-3′) were used for the amplification of a 85-bp region of the* B. longum* elongation factor Tu gene (*tuf*). The PCR amplification volume of 25 *μ*L contained 10 *μ*L of Fast SYBR Green Master Mix (Life Technologies), 5 *μ*L of DNA, 1 *μ*L of each 2.5 *μ*M primer, and 8 *μ*L of nuclease-free water. Duplicate qPCR amplifications were carried out consisting of a 20 s denaturation step at 95°C, followed by 40 cycles of 3 s at 95°C and 30 s at 60°C. Finally, viable cells/mL were obtained from the *C*_*t*_ values using the corresponding standard curve. The standard curve and detection limit were determined using a pure culture of* B. longum* CUETM 172. One mL of culture was serially diluted eight times in sterile reconstituted milk. Next, 1 mL of each dilution was treated with PMA as described before. DNA extraction and quantification were performed as for the TIM-1 samples. After qPCR amplification, *C*_*t*_ results were plotted against the corresponding viable count (CFU/mL).

The ORAC analysis was also performed as described before on fermented milk samples after dilution in a 1 : 500 ratio with phosphate buffer and on dialysate samples of the* in vitro* digestion experiments after dilution in a 1 : 50 ratio with phosphate buffer.

### 2.3. Statistical Analysis

All statistical analyses were performed using JMP version 9 Software (SAS Institute, Cary, NC, USA). ORAC values of the different bifidobacteria strains were compared with analysis of covariance (ANCOVA) with optical density at 600 nm as covariate. The means separation was done using the pairwise comparisons of least squares means using Student's *t*-tests (LSMeans Student's *t*). ORAC values of the nonfermented milk and the milk fermented by the five* B. longum* subsp.* longum* were compared with analysis of variance (ANOVA).

## 3. Results

### 3.1. Antioxidant Capacity of Cell-Free Extracts

ORAC results were weighted with the optical density at 600 nm as covariate, as there was a linear relationship between the ORAC values and this parameter (*F* = 38.2226; *P* < 0.0001) (Table 2). The ORAC values ranged between 76.5 ± 38.2 and 274.3 ± 38.4 *μ*mol TE/L and differed among species and strains (*F* = 2.2141; *P* = 0.0009). The pairwise comparisons divided the 38 strains into three groups. Three strains exhibited ORAC values higher than 250 *μ*mol TE/L, of which two strains CUETM 172 and CUETM 171 belong to* B. longum* subsp.* longum*. The last strain, CUETM 174, belongs to* B. catenulatum*.* B. longum* subsp.* infantis* ATCC 15702,* B. animalis* subsp.* lactis* BB-12, and* B. adolescentis* ATCC 15703 possessed the lowest antioxidant capacity (lower than 100 *μ*mol TE/L).

### 3.2. Genetic Analysis of Oxidative Stress Response Genes

**Figure 1:**
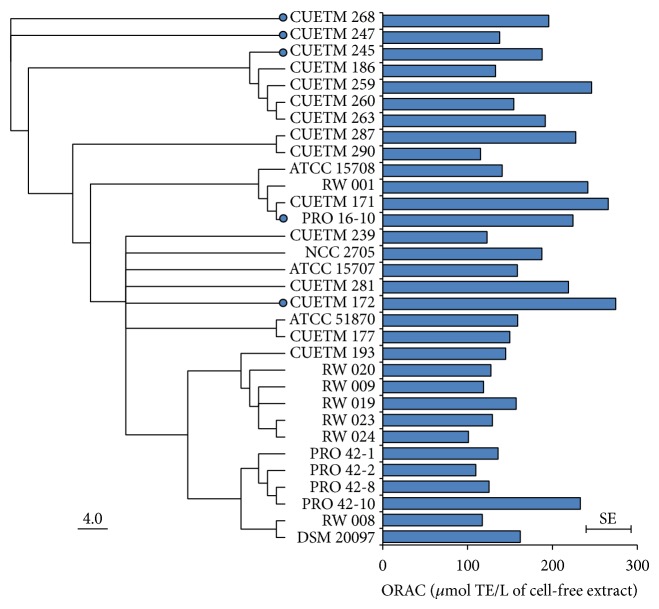
Antioxidant capacity of* B. longum* subsp.* longum* strains paired with the MLSA dendrogram. On the left, Jukes-Cantor neighbor-joining dendrogram constructed using the concatenated sequences of five loci (*mutT1, ahpC, trx, nrdA*, and* ppk*). Strains marked with a blue dot are the strains selected for milk fermentation. The length of the branches expressed in units of substitutions per site of the sequence alignment is indicated by the scale bar. On the right, oxygen radical absorbance capacity (ORAC) values correspond to the weighted means determined by ANCOVA. The error bar represents the standard error (SE).

MLSA based on five genes (*mutT1, ahpC, trx, nrdA,* and* ppk*), which are predicted to be involved in the oxidative stress response of bifidobacteria, was performed to evaluate the genetic diversity of the 32 tested* B. longum* subsp.* longum* strains. The allele numbers and ST numbers were determined for all strains (see Supplementary Material, Table S3). For the 32 strains, there are 22 different STs based on the concatenated sequences of the five sequenced loci, a total of 2,079 bp. Despite the high percent of identity (96.2%) of the concatenated sequences of the 32 strains, polymorphic nucleotides were found in all five genes (see Supplementary Material, Table S4). A phylogenetic tree of the concatenated sequences of the five loci for the 32* B. longum* subsp.* longum* strains was constructed and compared to the antioxidant capacities of these strains (Figure 1).* B. longum* subsp.* longum* CUETM 171 and CUETM 172, both having high ORAC values, did not belong to the same cluster in the phylogenetic tree. The allele for* ahpC* was the only allele the two strains had in common. The four* B. longum* subsp.* longum* PRO 42 strains, isolated from the same human donor, had the same ST number (Table S3), but three had low antioxidant capacity, while the value observed for PRO 42-10 was higher. Five strains spanning the varying antioxidant capacities and different genetic groups were selected to perform experiments with fermented milk (Figure 1). More details about strain selection are available in Supplementary Material, Table S5.

### 3.3. Dynamic* In Vitro* Gastrointestinal Digestion (TIM-1) of Fermented Milk

#### 3.3.1. Fermentation of Milk

All five strains (CUETM 172, CUETM 268, CUETM 245, CUETM 247, and PRO 16-10) acidified the milk until a mean pH of 4.5 and reached cell counts of 10^9^ CFU per mL.

#### 3.3.2. Bacterial Survival

**Figure 2:**
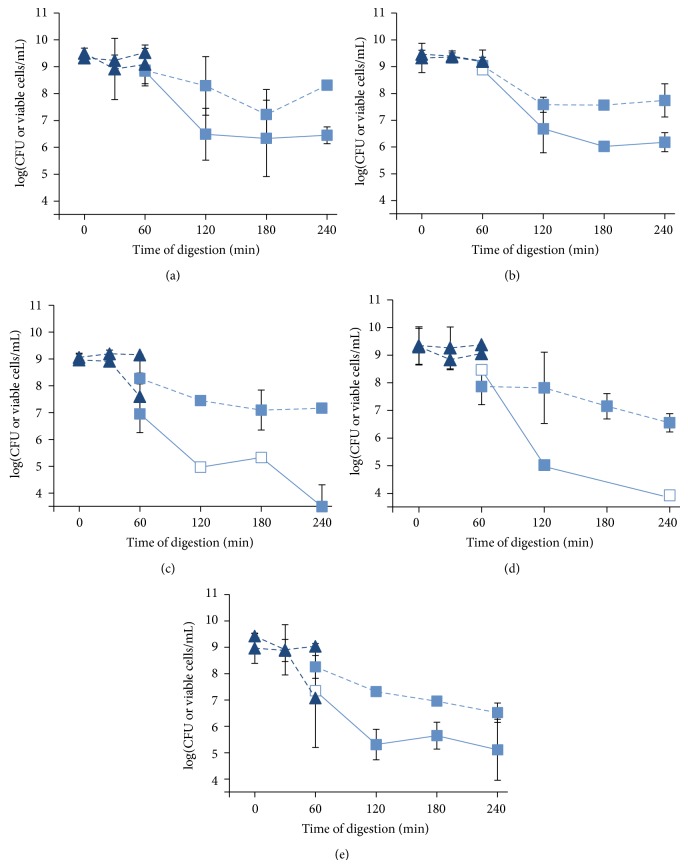
Survival curve during* in vitro* digestion (TIM-1) of fermented milk with* B. longum* subsp.* longum* CUETM 172 (a), CUETM 247 (b), CUETM 245 (c), CUETM 268 (d), and PRO 16-10 (e). The cell concentrations were determined by viable counts in CFU/mL (solid line) and by PMA-qPCR in viable cells/mL (dashed line). Samples were taken in gastric (▲) and duodenal (■) compartments. Empty symbols indicate that only one value was obtained. The limit of detection of PMA-qPCR was 3.51 log of viable cells/mL. The error bars represent the standard deviation.

During the first 30 min of digestion, viability of the five* B. longum* subsp.* longum* strains remained high (Figure 2). After 60 min, the viable cell counts decreased for CUETM 245 and PRO 16-10. However, the cell concentrations evaluated with PMA-qPCR remained stable over this period for all strains. After 120 min, the PMA-qPCR counts were higher than the viable counts (CFU/mL) in the duodenal compartment.* B. longum* subsp.* longum* CUETM 172, CUETM 247, and CUETM 245 showed the smallest decline in viability with a loss of about 1 log cells/mL between 60 and 240 min of digestion in the duodenal compartment.* B. longum* subsp.* longum* CUETM 268 and PRO 16-10 were more affected by the conditions of the duodenal compartment, as cell concentrations decreased from 8 log to 6.5 log of viable cells/mL.

**Figure 3:**
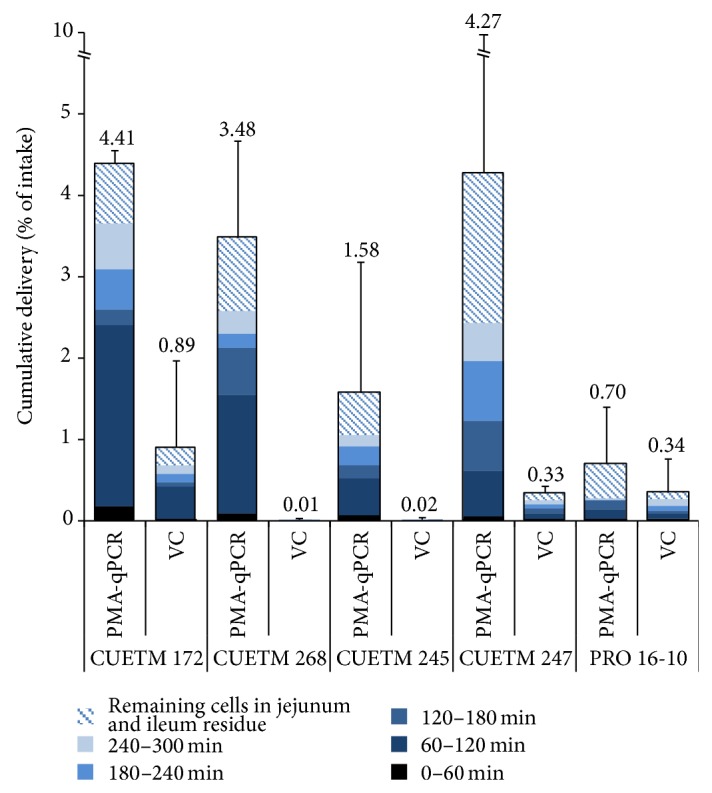
Survival rate of the five strains used for milk fermentation during* in vitro* digestion (TIM-1). The viable cells were determined by PMA-qPCR and by viable counts (VC) in the effluent after each hour of digestion. The remaining cells in the jejunum and ileum residue after 300 min of digestion were included in the survival rate. The results are presented relative to the total cells in the fermented milk at the start (% of intake). The error bars represent the standard deviation.

In the effluent, total number of cells evaluated with PMA-qPCR was at least 10^9^ viable cells for all strains (CUETM 172: 2.64 × 10^10^ cells, CUETM 268: 4.09 × 10^10^ cells, CUETM 245: 4.25 × 10^9^ cells, CUETM 247: 1.26 × 10^10^ cells, and PRO 16-10: 4.99 × 10^9^ cells). Survival rates of cells in the TIM-1 effluent estimated by PMA-qPCR were higher than those determined with viable counts (Figure 3).* B. longum* subsp.* longum* CUETM 172, 268, and 247 exhibited survival rates higher than 3% according to the PMA-qPCR results. In contrast, the survival rate of* B. longum* subsp.* longum* PRO 16-10 was lower than 1%.

#### 3.3.3. Bioaccessibility of Antioxidants in Fermented Milk

Before digestion (Table 3), there was no significant difference between the antioxidant capacity of nonfermented milk and milk fermented by each of the five bifidobacteria strains (*F* = 0.9870; *P* = 0.4649).

**Figure 4:**
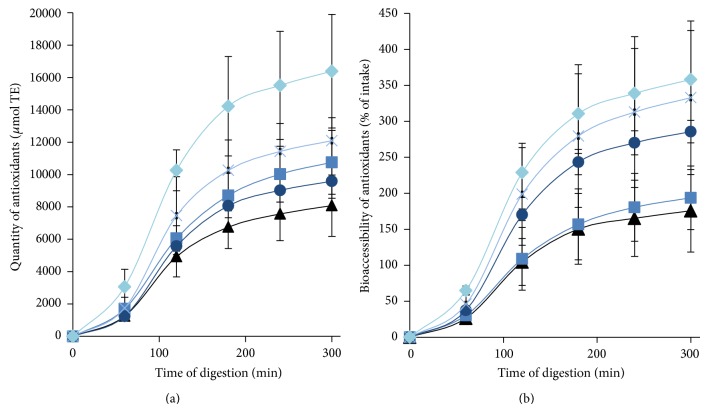
Bioaccessibility of antioxidants (in dialysates) evaluated with oxygen radical absorbance capacity (ORAC) during* in vitro* digestion (TIM-1) of fermented milk (300 g) by* B. longum* subsp.* longum* CUEMT 172 (▲), CUETM 268 (■), CUETM 245 (●), CUETM 247 (**X**), and PRO 16-10 (♦). (a) The cumulative quantity of bioaccessible antioxidants is expressed in *μ*mol Trolox equivalent (TE). (b) The bioaccessibility of antioxidants expressed as a percentage of intake (antioxidants in 300 g of fermented milk before digestion). The error bars represent the standard deviation.

During digestion, the antioxidant capacity remained higher in the jejunal compartment than the ileal compartment at each sampling point (data not shown). The quantity of bioaccessible antioxidants delivered was determined by multiplying the antioxidant capacity from the jejunal and ileal compartments at each hour of digestion by the volume of dialysate (Figure 4(a)). The largest delivery of antioxidants was obtained between 60 and 120 min of digestion in both jejunal and ileal compartments. After five hours of digestion, the milk fermented with* B. longum* subsp.* longum* PRO 16-10 showed the highest quantity of antioxidants at 16,383 *μ*mol TE. The lowest quantity of antioxidants (8,080 *μ*mol TE) was obtained by milk fermented with* B. longum* subsp.* longum* CUETM 172. Antioxidant bioaccessibility was expressed as a percentage of the intake of antioxidant in the meal (300 g of fermented milk) before digestion (Figure 4(b)). By the end of digestion, the antioxidants in fermented milk possessed a bioaccessibility ranging from 175% for* B. longum* subsp.* longum* CUETM 172 to 358% for* B. longum* subsp.* longum* PRO 16-10.

## 4. Discussion

As the antioxidant capacity of cell-free extracts of 32* B. longum* subsp.* longum* strains is highly strain specific, it is thus possible to classify bifidobacteria strains according to this characteristic. However, in the present study, the sequence types of five genes coding for responses to oxidative stress were not correlated with antioxidant capacity among these 32 strains. Although* B. longum* subsp.* longum* CUETM 172 showed the highest antioxidant capacity during the screening of 32* B. longum* subsp.* longum* strains, this was not reflected in the antioxidant capacity of the fermented milk. The antioxidant capacity of nonfermented milk and fermented milk in this study is similar to reconstituted milk (15% skim milk powder) [42] and a commercial UHT skimmed cow milk [43]. The development of radical scavengers during fermentation of milk can be explained in part by proteolysis [17], but bifidobacteria have low proteolytic activities [44, 45]. Indeed, antioxidant molecules can be located in the cytoplasm of bacteria [46]. If the cell membrane is intact, the antioxidant capacity of these molecules will not be detected with the ORAC assay. Even though the antioxidant capacity of the fermented milk before digestion is lower than blueberries and red wine (Table 3), this does not mean that they are less suitable sources of antioxidants. The quantity of bioaccessible antioxidant compounds is variable in foods such as fruit and vegetables [27]. For instance, the total bioaccessibility of anthocyanins in wild blueberries during TIM-1 digestion was less than 10% of the intake [28]. Furthermore, Lila et al. [28] have shown that bioaccessibility data overestimate* in vivo* (rodent) bioavailability, since TIM-1 hollow fibres for dialysis do not perfectly simulate the endothelial cells of the GIT. Moreover, the bioavailability of antioxidants is affected by many factors, such as food microstructure and chemical interactions with other phytochemicals and biomolecules [27]. In the future, antioxidants produced by bacteria such as* B. longum* subsp.* longum* strains will need to be tested* in vivo* in order to evaluate whether the antioxidants are absorbed in the same way as in the TIM-1 model and whether they are metabolized or not.

We hypothesize that the bioaccessibility of antioxidants produced by* B. longum* subsp.* longum* could be improved by the harsh conditions of the GIT. These conditions can stress or kill bifidobacteria present in the fermented milk, even though* B. longum* strains are well adapted to the colon ecosystem [13]. However, it is difficult to evaluate the difference of these two states with viable counts because stress can lead to viable but noncultivable cells (VBNC state) [47]. The PMA-qPCR method can enumerate both viable and VBNC cells [48]. The five strains were not affected by the high acidity of the stomach compartment in the TIM-1 for 60 min, according to viable counts and PMA-qPCR results. All five* B. longum* subsp.* longum* strains were affected to varying degrees by the bile salts in the duodenum compartment, despite the presence in the genome of* B. longum* of the* bsh* gene encoding a bile salt hydrolase [49]. As for acid tolerance, resistance to bile salts seems to be a strain-specific characteristic and together they have a major influence on the final survival rate through the GIT [12]. Saide and Gilliland [15] have in fact suggested that the encounter with bile could improve the delivery of antioxidants to the intestine.

Data on pharmacokinetics of bifidobacteria in different parts of the intestinal tract and in colon simulation models are mainly based on comparison of bacterial strains before and after ingestion rather than on precise data on bacterial survival rates [50].* Bifidobacterium* sp. can survive transit through the intestinal tract with recovery rates in faeces ranging from 20 to 22% for the fermented milk and lyophilized form, respectively [51, 52]. Among bifidobacteria,* B. animalis* subsp.* lactis* strains displayed the highest survival rates during* in vivo* ileal perfusion and simulated gastric transit with an estimated survival rate ranging from 23.5% to 37.5% [53, 54] with a faecal recuperation of 30% [55]. Only single strains of* B. longum* subsp.* longum* (LMG 13196) exhibited survival rates comparable with those observed for the* B. animalis* subsp.* lactis* strains during* in vitro* assessment of the transit tolerance [56]. Fujiwara et al. [57] noted that* B. longum* subsp.* longum* SBT2928 was found in good proportions in the faeces.

The survival rate obtained in this study can best be compared to other studies using dairy products as a delivery mode for probiotics in TIM-1 as milk is known to provide protection to probiotic bacteria [58]. The survival of the five* B. longum* subsp.* longum* strains determined by viable counts is very low (0.8–0.01%) compared to* Lactobacillus amylovorus* DSM 16698 (survival rate up to 100%) [32].* Bifidobacterium bifidum*,* L. acidophilus*, and* Pediococcus acidilactici* UL5 have also demonstrated better survival rates (10–20%) [11, 31]. The survival rates of the five* B. longum* subsp.* longum* strains seem to be more comparable to those of* Lactococcus lactis* ATCC 11454 (0.00073%) [31],* Streptococcus thermophilus* ST20, and* Lactobacillus delbrueckii* subsp.* bulgaricus* LB9 (close to the detection limit) [11]. However, the results presented here show that viable counts underestimate cell survival and* in vivo* the presence of other food components could enhance protection of the bacteria.

Without the use of PMA-qPCR, we would assume that all five strains in this study had a low survival rate. However, the VBNC state is revealed by the difference between PMA-qPCR estimates and viable counts. For* B. longum* subsp.* longum* PRO 16-10, the absence of difference between PMA-qPCR and viable cell counts indicates that cells did not reach the VBNC state and only a small portion survived after digestion in the TIM-1. Adams [59] suggested that variable amounts of dead cells might contribute to the differences in effects observed when administering live probiotics. Even though some probiotics have low survival rates, the number of cells that would reach the colon alive may be sufficient. For milk fermented by all five strains in this study, there was a greater amount of antioxidants present in the dialysate than in the milk before digestion (1.5–3.5-fold higher). For* B. longum* subsp.* longum* PRO 16-10, the quantity of bioaccessible antioxidants delivered by the fermented milk was higher at the end of digestion, which was accompanied by a low survival rate (0.70%).

The evaluation of antioxidant capacity in cell-free extracts must be complemented by cell survival assays in order to properly select strains for fermentation of milk with the best bioaccessibility of antioxidants. This is the first time that strains with low survival rate in fermented milk are shown to deliver more bioaccessible antioxidants during* in vitro* dynamic digestion. In addition to the liberation of antioxidants, dead bacteria provide other health benefits such as immunomodulation and anti-inflammatory effects [60–62]. In order to provide other kinds of benefit to the host, it is still important to ensure that a portion of the intake of probiotics survive the GIT passage. It has been suggested that the antioxidant effect from probiotics reaching the colon can be explained by the scavenging of oxidant compounds or the prevention of their generation in the colon [62]. However, the presence of antioxidants in the dialysate suggests that a major portion of antioxidants produced by* B. longum* strains may be absorbed in the small intestine and could thus be transported in the blood.

## 5. Conclusion

Milk fermented by different strains of* B. longum* subsp.* longum* provided bioaccessible antioxidants during digestion. However, the characterization of antioxidant capacity of cell-free extracts cannot be used as a selection criterion for antioxidant probiotic strains because survival rate in the GIT had more influence on the bioaccessibility of antioxidants. The improved bioaccessibility probably comes from the death of a portion of* B. longum* subsp.* longum* cells. The milk fermented with the strain with the lowest survival rate in the upper GIT (*B. longum* subsp.* longum* PRO 16-10) had the highest bioaccessibility of antioxidants. On the contrary, the milk fermented with the strain with the best survival rate (*B. longum* subsp.* longum* CUETM 172) had the lowest bioaccessibility of antioxidants. Probiotics are usually defined as “live microorganisms, which, when administered in adequate amounts, confer a health benefit on the host” (FAO/WHO) but variable amounts of dead cells during digestion of fermented milk may contribute to health benefits by providing bioaccessible antioxidants. These antioxidants could lead to the improving antioxidant capacity of human blood.

## Supplementary Material

The supplementary material includes the primers used in PCR (Table S1), the parameters of TIM-1 in vitro digestion model (Table S2) as well as the results of the Multilocus Sequence Analysis of Bifidobacterium strains (Tables S3 to S5).

## Figures and Tables

**Table 1:** Origin of *Bifidobacterium longum* subsp*.  longum* strains.

Strain	Origin	Reference or source
ATCC 15707	Adult intestine	American Type Culture Collection, Manassas, VA
ATCC 15708	Child feces	American Type Culture Collection, Manassas, VA
ATCC 51870	Child feces	American Type Culture Collection, Manassas, VA
DSM 20097	Calf feces	Deutsche Sammlung von Mikroorganismen und Zellkulturen GmbH, Braunschweig, Germany)
NCC 2705	Infant feces	Nestlé, Lausanne, Switzerland
CUETM 171	Child feces	Bahaka et al. [63]
CUETM 172	NA^1^	Bahaka et al. [63]
CUETM 177	Child feces	Bahaka et al. [63]
CUETM 186	Child feces	Bahaka et al. [63]
CUETM 193	Child feces	Bahaka et al. [63]
CUETM 239	Child feces	Bahaka et al. [63]
CUETM 245	Child feces	Bahaka et al. [63]
CUETM 247	Child feces	Bahaka et al. [63]
CUETM 259	Child feces	Bahaka et al. [63]
CUETM 260	Child feces	Bahaka et al. [63]
CUETM 263	Child feces	Bahaka et al. [63]
CUETM 268	Child feces	Bahaka et al. [63]
CUETM 281	Child feces	Bahaka et al. [63]
CUETM 287	Child feces	Bahaka et al. [63]
CUETM 290	Child feces	Bahaka et al. [63]
PRO 16-10	Adult feces	Savard et al. [64]
PRO 42-1	Adult feces	Savard et al. [64]
PRO 42-10	Adult feces	Savard et al. [64]
PRO 42-2	Adult feces	Savard et al. [64]
PRO 42-8	Adult feces	Savard et al. [64]
RW 001	Commercial preparation	Roy et al. [65]
RW 008	Commercial preparation	Roy et al. [65]
RW 009	Commercial preparation	Roy et al. [65]
RW 019	Commercial preparation	Roy et al. [65]
RW 020	Commercial preparation	Roy et al. [65]
RW 023	Commercial preparation	Roy et al. [65]
RW 024	Commercial preparation	Roy et al. [65]

^1^Not available.

**Table 2:** Antioxidant capacity of cell-free extracts evaluated by the oxygen radical absorbance capacity (ORAC) assay.

Genus and species	Strain	ORAC (*µ*M TE^1^) ± SE^2^
*B* ^3^ *. adolescentis *	ATCC 15703	76.5 ± 38.2^M^
*B* ^3^ *. animalis *subsp.* lactis *	BB-12	79.8 ± 38.4^LM^
*B. longum *subsp.* infantis *	ATCC 15702	85.9 ± 38.9^KLM^
*B. longum *subsp.* longum *	RW 024	101.0 ± 39.7^JKLM^
*B. longum *subsp.* longum *	PRO 42-2	109.5 ± 39.1^IJKLM^
*B. longum *subsp.* longum *	CUETM 290	115.0 ± 38.2^HIIJKLM^
*B. longum *subsp.* longum *	RW 008	117.0 ± 33.1^IIJKLM^
*B. longum *subsp.* longum *	RW 009	118.7 ± 39.2^HIIJKLM^
*B. longum *subsp.* longum *	CUETM 239	122.7 ± 33.6^HIIJKLM^
*B. longum *subsp.* longum *	PRO 42-8	125.0 ± 38.2^GHIIJKLM^
*B. longum *subsp.* longum *	RW 020	127.5 ± 39.1^FGHIIJKLM^
*B. longum *subsp.* longum *	RW 023	129.2 ± 40.1^EFGHIIJKLM^
*B. longum *subsp.* longum *	CUETM 186	132.4 ± 38.6^DEFGHIIJKLM^
*B. longum *subsp.* longum *	PRO 42-1	135.6 ± 38.1^DEFGHIIJKLM^
*B. longum *subsp.* longum *	CUETM 247	137.5 ± 38.0^DEFGHIIJKLM^
*B. longum *subsp.* longum *	ATCC 15708	140.5 ± 12.5^IJKLM^
*B. longum *subsp.* longum *	CUETM 193	144.4 ± 39.1^CDEFGHIJKLM^
*B. longum *subsp.* longum *	CUETM 177	149.2 ± 38.5^CDEFGHIJKLM^
*B. longum *subsp.* longum *	CUETM 260	154.1 ± 33.1^CDEFGHIJKLM^
*B. longum *subsp.* longum *	RW 019	157.2 ± 39.0^BCDEFGHIJKLM^
*B. longum *subsp.* longum *	ATCC 15707	158.2 ± 38.5^BDEFGHIJKLM^
*B. longum *subsp.* longum *	ATCC 51870	158.5 ± 39.5^BCDEFGHIJKLM^
*B. longum *subsp.* longum *	DSM 20097	162.0 ± 38.6^BCDEFGHIJKLM^
*B. longum *subsp.* suis *	ATCC 27533	175.7 ± 38.2^ABCDEFGHIJKLM^
*B. longum *subsp.* longum *	NCC 2705	187.3 ± 38.3^ABCDEFGHIJKL^
*B. longum *subsp.* longum *	CUETM 245	187.4 ± 33.1^ABCDEFGHIJ^
*B. longum *subsp.* longum *	CUETM 263	191.0 ± 38.5^ABCDEFGHIJK^
*B. longum *subsp.* longum *	CUETM 268	195.3 ± 38.1^ABCDEFGHIJ^
*B. longum *subsp.* longum *	CUETM 281	218.2 ± 39.5^ABCDEFGHI^
*B. longum *subsp.* longum *	PRO 16-10	223.5 ± 39.1^ABCDEFGH^
*B. longum *subsp.* longum *	CUETM 287	227.1 ± 38.0^ABCDEFG^
*B. longum *subsp.* longum *	PRO 42-10	232.6 ± 38.1^ABCDEF^
*B* ^3^ *. breve *	ATCC 15698	237.0 ± 38.1^ABCDE^
*B. longum *subsp.* longum *	RW 001	241.5 ± 39.0^ABCD^
*B. longum *subsp.* longum *	CUETM 259	245.7 ± 38.1^ABC^
*B. longum *subsp.* longum *	CUETM 171	265.1 ± 39.1^AB^
*B* ^3^ *. catenulatum *	CUETM 174	266.4 ± 33.5^A^
*B. longum *subsp.* longum *	CUETM 172	274.3 ± 38.4^A^

Means with different capital letter superscripts were significantly different (*P* < 0.05).

^1^Trolox equivalent.

^2^Results were expressed as means ± standard error (*n* = 3).

^3^ *Bifidobacterium*.

**Table 3:** Comparison of antioxidant activity (ORAC) for a portion of 100 g of different food types.

Food description	ORAC value (*µ*mol TE^1^/100 g)
Blueberries, wild, raw^2^	9621
Wine, table, red, Cabernet Sauvignon^2^	4523
Cranberry juice, unsweetened^2^	1452
Fermented milk (CUETM 245)	1318
Fermented milk (PRO 16-10)	1312
Fermented milk (CUETM 247)	1255
Fermented milk (CUETM 268)	1175
Fermented milk (CUETM 172)	1076
Nonfermented milk	1174
Commercial UHT skimmed cow milk^3^	1270
Apple juice, canned or bottled, unsweetened, without added ascorbic acid^3^	414

^1^Trolox equivalent.

^2^Haytowitz and Bhagwat [66].

^3^Zulueta et al. [43].
