# Supplementary material for: Bioaccessible Antioxidants in Milk Fermented by Bifidobacterium longum subsp. longum Strains
Source: Biomed Res Int. 2015 Feb 23;2015:169381. doi: 10.1155/2015/169381 (PMC4352726; doi:10.1155/2015/169381)
Supplement: Supplementary file 1 — The supplementary material includes the primers used in PCR (Table S1), the parameters of TIM-1 in vitro digestion model (Table S2) as well as the results of the Multilocus Sequence Analysis of Bifidobacterium strains (Tables S3 to S5). [file 169381.f1.pdf]

Supplementary material

Table S1. Primers used for multilocus sequence analysis (MLSA) of *B. longum* subsp. *longum* strains.

Locus	Gene position[1]	Sequence 5'→3'	Amplicon size (bp)
mutT1	839309-840483	TCGAAATGGCGCTGCTCCCG<br>ATCGACATGGACGTGCCCGC	446
ahpC	1610493-1611056	GTCCAGTTGGCGGGGCAGAC<br>CCCGGCCGACTTCACCTTCG	396
trx	1648290-1649309	ATGCCTGGCGCACTGGTCAC<br>CTGGCTACACGGCCGCCATC	868
nrdA	1672349-1674544	TTCGAACAGCGCACGGACCC<br>ACCTCAACGCCCACCACCCT	905
ppk	1878102-1880339	CCACGCGATGTTGGCGTCCT<br>TTCGCCGCGATCTTCGCCTC	1152

[1]Gene position on genome of *B. longum* subsp. *longum* BBMN68 (GenBank accession number CP002286)

Table S2. Description of the four compartments of the TIM-1 *in vitro* digestion model.

Compartment	Initial content (I), secretions (S) and dialysis fluid (D)	Volume (mL)	*t½*	*β*	*t* (min)	pH
Stomach	I: Gastric juice (10 mL, pH 2): 2080 U/mL of pepsin and 80 U/mL of lipase in gastric electrolyte solution (NaCl 6.2 g/L, KCl 2.2 g/L, $CaCl_2$ 0.3 g/L, $NaHCO_3$ 1.5 g/L)<br><br>S: Gastric juice (pH 4): 0.25 mL/min<br>Water or HCl 1M: 0.25 mL/min	310	70	2	0<br>10<br>20<br>40<br>60<br>90<br>120<br>300	5.5<br>5.0<br>4.2<br>2.8<br>2.1<br>1.8<br>1.7<br>1.7
Duodenum	I: 15 g of 21% pancreatin solution, 30 g of 4 % porcine bile solution, 1 mL of 2 mg/mL trypsin solution and 15 g of small intestine electrolyte solution (pH 7) (NaCl 5.0 g/L, KCl 0.6 g/L, $CaCl_2$ 0.3 g/L)<br><br>S: 21 % pancreatin solution: 0.25 mL/min<br>4 % bile solution (first 30 min) then 2 %: 0.25 mL/min	55	160	1.6		6.3
Jejunum	I: Small intestine electrolyte solution<br>S:$NaHCO_3$ 1M if necessary<br>D:Small intestine electrolyte solution: 10 mL/min	100				6.5
Ileum	I:Small intestine electrolyte solution<br>S:$NaHCO_3$ 1M if necessary<br>D:Small intestine electrolyte solution: 10 mL/min	100				7.4

Gastric and ileal deliveries are modeled with the power exponential formula: $f = 1-2^{-(t/t1/2)\beta}$, where *f* represents the fraction of meal delivered, *t* the time of delivery, *t½* the half-time of delivery, and *β* a coefficient describing the shape of the curve. All reagents

were from Sigma-Aldrich (Oakville, ON, Canada) except lipase which was kindly provided by Amano enzyme USA (Elgin, IL, USA).

Table S3. Typing of 32 strains of *B. longum* subsp. *longum* by multilocus sequence analysis (MLSA) of five loci.

Strain	Clonal group[1]	ST[2]	Allele code				
			mutT1	*ahpC*	*trx*	*nrda*	*ppk*
CUETM 239		1	1	5	3	5	1
CUETM 171		7	8	5	10	9	3
PRO 16-10		8	9	3	11	3	1
CUETM 287		11	3	2	3	5	4
ATCC 15708		13	2	4	5	4	2
RW008		14	2	3	6	2	2
CUETM 290		15	5	2	7	7	3
ATCC 15707		17	2	3	3	4	5
CUETM 193	1	2	2	1	4	3	2
CUETM 177	1	3	3	1	4	5	2
RW020	1	4	2	6	3	3	2
CUETM 186	1	5	2	2	2	1	2
CUETM 245	1	5	2	2	2	1	2
CUETM 259	1	5	2	2	2	1	2
CUETM 260	1	5	2	2	2	1	2
DSM 20097	1	6	7	1	3	2	2
PRO 42-1	1	9	2	1	3	1	2
PRO 42-10	1	9	2	1	3	1	2
PRO 42-2	1	9	2	1	3	1	2
PRO 42-8	1	9	2	1	3	1	2
RW009	1	10	2	1	1	3	2
RW019	1	10	2	1	1	3	2
RW023	1	10	2	1	1	3	2
RW024	1	10	2	1	1	3	2
CUETM 247	1	16	2	2	8	6	2
CUETM 268	1	16	2	2	8	6	2
CUETM 263	1	18	2	2	2	8	2
ATCC 51870	1	19	3	1	3	3	2
RW001	1	20	2	1	9	5	2
CUETM 172	1	22	2	5	3	3	2
NCC 2705	2	12	4	3	4	4	2
CUETM 281	2	21	6	3	4	4	1

[1] The clonal groups were determined with the START2 software.

[2] Sequence type

Table S4. Multilocus sequence analysis (MLSA) of five genes related to oxidative stress response.

Gene	Fragment analyzed (bp)	No. of alleles	No. of polymorphic sites	π (nucleotide diversity)[1]	d_N[2]	d_S[3]	d_N/d_S[4]	I^S_A[4]
mutT1	369	9	12	0.00916	0.0191	0	-	
ahpC	287	6	25	0.003376	0.0142	0.1138	0.1248	
trx	558	11	23	0.00944	0.0064	0.0541	0.1185	0.18
nrdA	526	9	14	0.00687	0.0113	0.0053	2.1407	
ppk	339	5	6	0.00367	0.0128	0	-	

[1] The nucleotide diversity was determined with the DnaSP v5.10.1 software.

[2]No. of substitutions per non-synonymous site

[3]No. of substitutions per synonymous site

[4] The standardized index of association (I^S_A) and the ratios of substitution per non-synonymous site to substitution per synonymous site (dN/dS) were determined with the START2 software.

MLSA of *B. longum* subsp. *longum*

The nucleotide diversity (π) of the five loci ranged between 0.00367 (*ppk*) and 0.03376 (*ahpC*). Two clonal groups were found. The first clonal group (consisting of 22 *B. longum* subsp. *longum* strains) contained 12 of the 22 ST profiles. Only two strains are in the second clonal group (NCC 2705 and CUETM 281). The d_N/d_S of *ahpC* and *trx* were lower than 1, indicating selection against amino acid changes. In contrast, the sequence of *ppk* and *mutT1* revealed absence of

synonymous substitutions and the d_N/d_S of *nrdA* was higher than one. I^S_A of the concatenated sequences of all five loci was near zero (0.18), confirming the linkage disequilibrium of the genetic variations in the five oxidative stress response genes.

Table S5. Groups of *B. longum* subsp. *longum* strains determined from the MLSA dendrogram (Figure1). Strains in bold were selected to monitor their growth and obtain curves.

Group	1	2	3	4	5	6
Strain	**CUETM 268**	**CUETM 247**	**CUETM 245**<br>CUETM 186<br>CUETM 259<br>CUETM 260<br>CUETM 263	CUETM 287<br>**CUETM 290**	ATCC 15708<br>RW 001<br>**CUETM 171**<br>**PRO 16-10**	CUETM 239<br>**NCC 2705**<br>ATCC 15707<br>CUETM 281<br>**CUETM 172**<br>ATCC 51870<br>CUETM 177<br>CUETM 193<br>**RW 020**<br>RW 019<br>RW 009<br>RW 023<br>RW 024<br>PRO 42-1<br>**PRO 42-2**<br>PRO 42-8<br>PRO 42-10<br>RW 008<br>DSM 20097

Growth of ten *B. longum* subsp. *longum* strains (Table S5) in MRS supplemented with lactose was monitored as a preliminary step to *in vitro* digestion of fermented milk to ensure that selected strains would grow in milk. One strain in each group was at least selected. The strains CUETM 171 and CUETM 172 were chosen for their high antioxidant capacity, while CUETM 290 and PRO 42-2 were chosen for their low antioxidant capacity. The strains CUETM 245 and RW 020 represented the strains with moderate antioxidant capacity. The strains, NCC 2705 was chosen as this strain was the first *B. longum* strain whose

genome was completely sequenced. Finally, at least two strains isolated from healthy adult feces were chosen, so PRO 16-10 was selected in addition to PRO 42-2 which was already selected.

These strains were pre-cultured in MRS-based broth (MRS without glucose; Rosell Institute, Montreal, QC, Canada) supplemented with 0.05% cysteine, 0.1% Tween 80, and 0.5% dextrose (EMD Chemicals, Fisher Scientific, Ottawa, ON, Canada) by adding 2% of the frozen stock culture. After 24 h of incubation at 37 °C in a glove box anaerobic chamber, 1% of the first pre-culture was added to the MRS supplemented with 0.5% lactose (EMD Chemicals) instead of dextrose and incubated for another 24 h at 37 °C. After two pre-cultures, 1 % was added to 10 mL of MRS-based broth supplemented with lactose and incubated for 24 h at 37°C. During the first 24 h of growth optical densities at 600 nm, viable counts, and pH values were determined seven times. Then, five strains (CUETM 172, CUETM 245, CUETM 247, CUETM 268, and PRO 16-10) that grew well, *i.e.* the cells reached 10^9 CFU per mL after 16 h of fermentation (data not shown) were chosen for fermenting reconstituted skim milk for the TIM-1 experiments.
